# 3-Benzyl-5-butyl-1,3,5-thia­diazinane-2-thione

**DOI:** 10.1107/S1600536809003882

**Published:** 2009-02-06

**Authors:** Mohammad Arfan, M. Nawaz Tahir, Muhammad Ishaq Ali Shah, Mohammad S. Iqbal

**Affiliations:** a Institute of Chemical Sciences, University of Peshawar, Peshawar 25120, Pakistan; b Department of Physics, University of Sargodha, Sargodha, Pakistan; c Department of Chemistry, Government College University, Lahore, Pakistan

## Abstract

In the title compound, C_14_H_20_N_2_S_2_, the 1,3,5-thia­diazinane-2-thione ring adopts an envelope conformation. The S=C bond length is 1.6776 (15) Å, whereas the S—C bond lengths are 1.7470 (15) and 1.8479 (17) Å. The intramolecular C—H⋯S hydrogen bond between the thione and the benzyl units along with the C—H⋯π interaction between the butyl group and the centroid of the benzene ring may be effective in stabilizing the molecule.

## Related literature

For the synthesis of the 1,3,5-thia­diazinane-2-thione nucleus, see: Aboul-fadi *et al*. (2002 ▶); Ertan *et al.* (1991 ▶, 1996 ▶). For its biological activity, see: Coro *et al.* (2005 ▶). For a related structure, see: Perez *et al.* (2001 ▶). For bond-length data, see: Allen (2002 ▶);
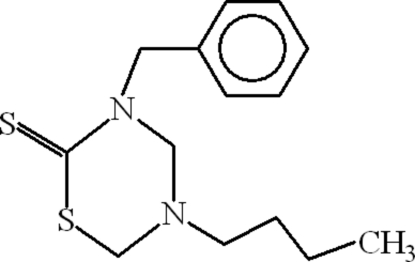


## Experimental

### 

#### Crystal data

C_14_H_20_N_2_S_2_
*M* *_r_* = 280.44
Triclinic, 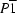

*a* = 7.6559 (2) Å
*b* = 9.9586 (3) Å
*c* = 11.1531 (4) Å
α = 66.917 (2)°
β = 70.649 (1)°
γ = 76.076 (2)°
*V* = 732.03 (4) Å^3^
*Z* = 2
Mo *K*α radiation
μ = 0.35 mm^−1^
*T* = 296 (2) K
0.26 × 0.20 × 0.18 mm

#### Data collection

Bruker Kappa APEXII CCD diffractometer
Absorption correction: multi-scan (*SADABS*; Bruker, 2005 ▶) *T*_min_ = 0.922, *T*_max_ = 0.942
15790 measured reflections
3759 independent reflections
2990 reflections with *I* > 2σ(*I*)
*R*_int_ = 0.025

#### Refinement

*R*[*F* ^2^ > 2σ(*F* ^2^)] = 0.035
*wR*(*F* ^2^) = 0.100
*S* = 1.03
3759 reflections
163 parameters
H-atom parameters constrained
Δρ_max_ = 0.22 e Å^−3^
Δρ_min_ = −0.20 e Å^−3^

### 

Data collection: *APEX2* (Bruker, 2007 ▶); cell refinement: *APEX2*; data reduction: *SAINT* (Bruker, 2007 ▶); program(s) used to solve structure: *SHELXS97* (Sheldrick, 2008 ▶); program(s) used to refine structure: *SHELXL97* (Sheldrick, 2008 ▶); molecular graphics: *ORTEP-3 for Windows* (Farrugia, 1997 ▶) and *PLATON* (Spek, 2003 ▶); software used to prepare material for publication: *WinGX* (Farrugia, 1999 ▶) and *PLATON* (Spek, 2003 ▶).

## Supplementary Material

Crystal structure: contains datablocks global, I. DOI: 10.1107/S1600536809003882/at2719sup1.cif

Structure factors: contains datablocks I. DOI: 10.1107/S1600536809003882/at2719Isup2.hkl

Additional supplementary materials:  crystallographic information; 3D view; checkCIF report

## Figures and Tables

**Table 1:** Hydrogen-bond geometry (Å, °)

*D*—H⋯*A*	*D*—H	H⋯*A*	*D*⋯*A*	*D*—H⋯*A*
C7—H7*B*⋯S1	0.97	2.60	3.0978 (16)	112
C12—H12*B*⋯CgA	0.97	2.96	3.7937 (19)	145

*CgA* is the centroid of the C1–C6 ring.

**Fig. 1.**
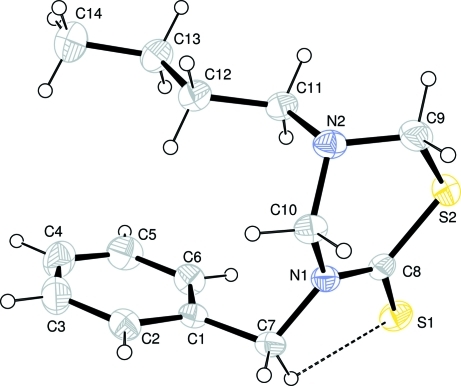
ORTEP drawing of the title compound, with the atom numbering scheme. The thermal ellipsoids are drawn at the 30% probability level. H-atoms are shown by small circles of arbitrary radii. The intramolecular H-bonding is shown by dotted lines.

## supplementary crystallographic information

### Comment

1,3,5-Thiadiazinane-2-thione nucleus is an important pharmacophoric nucleus and large number of its analogs have been synthesized through different synthetic approaches, including amines and carbon disulfide in aqueous KOH, *via* diathiocarbamate salt intermediate (Ertan *et al.*, 1991), from isothiocyanates and amines (Ertan *et al.*, 1996), and resin supported solid phase organic synthesis (Aboul-fadi *et al.*, 2002). Diverse bioactivities including antibacterial, antifungal, antimycobactarial, antitubercular, antiprotozoal, leishmanicidal, nematocidal and antiviral are reported for this nucleus in the literature (Coro *et al.*, 2005).

The crystal structure of 5-(2-carboxyethyl)-3-(fur-2-ylmethyl)-tetrahydro- 2*H*-1,3,5-thiadiazine-2-thione (Perez *et al.*, 2001) contains the same heterocyclic ring as the title compound (I), (Fig 1). The heterocyclic ring is in envelop form with the group (N1/C8/S2/C10/C9) in plane and the N2 displaced by -0.6778 (17) Å from it. The dihedral angle between the benzene ring A(C1—C6) and this group is 81.22 (5)°. The CCDC search (Allen *et al.*, 2002) showed that there are very few crystal structures having 1,3,5-thiadiazinane-2-thione nucleus, so some important bond lengths and bond angles are given in Table 1.

There are no indications of intermolecular contacts, however some weak intramolecular H-bonding is given in Table 2 [CgA is a centroid of the phenyl ring C1–C6].

### Experimental

The 1,3,5-thiadizinane thione was synthesized following the synthetic procedure reported by Ertan *et al.*, 1991. Carbon disulfide (20 mmol) was added portion-wise to a magnetically stirred solution of benzylamine (2.18 ml, 20 mmol) and potassium hydroxide (20 mmol) in 30 ml of water. The contents were stirred for 4 h at room temperature. Formaldehyde (37%, 40 mmol) was added to the reaction mixture and stirred for further 1 h. The reaction content was filtered and the filtrate was added drop-wise to a suspension of n-butylamine (1.97 ml, 20 mmol) to the phosphate buffer (pH 7.8) and stirred for 1 h at ambient temperature. The filterate of the reaction mixture was exhaustively extracted with dichloromethane. The aqueous reaction content was acidified with 15% HCl. The precipitated product was filtered off under suction and thoroughly washed with water. The air dried product was re-crystallized from ethanol. A colourless crystalline product [yield: 76%, m.p.: 381–383 K] was obtained.

### Refinement

H-atoms were positioned geometrically, with C—H = 0.93, 0.96 and 0.97 Å for aromatic, methyl and methylene H, and constrained to ride on their parent atoms, with *U*_iso_(H) = *xU*_eq_(C), where *x* = 1.5 for methyl H, and *x* = 1.2 for all other H atoms.

### Crystal data

**Table:**

C_14_H_20_N_2_S_2_	*Z* = 2
*M**_r_* = 280.44	*F*(000) = 300
Triclinic, *P*1	*D*_x_ = 1.272 Mg m^−^^3^
Hall symbol: -P 1	Mo *K*α radiation, λ = 0.71073 Å
*a* = 7.6559 (2) Å	Cell parameters from 2990 reflections
*b* = 9.9586 (3) Å	θ = 2.1–28.7°
*c* = 11.1531 (4) Å	µ = 0.35 mm^−^^1^
α = 66.917 (2)°	*T* = 296 K
β = 70.649 (1)°	Prismatic, colourless
γ = 76.076 (2)°	0.26 × 0.20 × 0.18 mm
*V* = 732.03 (4) Å^3^	

### Data collection

**Table:**

Bruker Kappa APEXII CCD diffractometer	3759 independent reflections
Radiation source: fine-focus sealed tube	2990 reflections with *I* > 2σ(*I*)
graphite	*R*_int_ = 0.025
Detector resolution: 7.40 pixels mm^-1^	θ_max_ = 28.7°, θ_min_ = 2.1°
ω scans	*h* = −10→10
Absorption correction: multi-scan (*SADABS*; Bruker, 2005)	*k* = −12→13
*T*_min_ = 0.922, *T*_max_ = 0.942	*l* = −15→14
15790 measured reflections	

### Refinement

**Table:**

Refinement on *F*^2^	Primary atom site location: structure-invariant direct methods
Least-squares matrix: full	Secondary atom site location: difference Fourier map
*R*[*F*^2^ > 2σ(*F*^2^)] = 0.035	Hydrogen site location: inferred from neighbouring sites
*wR*(*F*^2^) = 0.100	H-atom parameters constrained
*S* = 1.03	*w* = 1/[σ^2^(*F*_o_^2^) + (0.0507*P*)^2^ + 0.1133*P*] where *P* = (*F*_o_^2^ + 2*F*_c_^2^)/3
3759 reflections	(Δ/σ)_max_ < 0.001
163 parameters	Δρ_max_ = 0.22 e Å^−^^3^
0 restraints	Δρ_min_ = −0.20 e Å^−^^3^

### Special details

**Table:**

Geometry. Bond distances, angles *etc*. have been calculated using the rounded fractional coordinates. All su's are estimated from the variances of the (full) variance-covariance matrix. The cell e.s.d.'s are taken into account in the estimation of distances, angles and torsion angles
Refinement. Refinement of *F*^2^ against ALL reflections. The weighted *R*-factor *wR* and goodness of fit *S* are based on *F*^2^, conventional *R*-factors *R* are based on *F*, with *F* set to zero for negative *F*^2^. The threshold expression of *F*^2^ > σ(*F*^2^) is used only for calculating *R*-factors(gt) *etc*. and is not relevant to the choice of reflections for refinement. *R*-factors based on *F*^2^ are statistically about twice as large as those based on *F*, and *R*- factors based on ALL data will be even larger.

### Fractional atomic coordinates and isotropic or equivalent isotropic displacement parameters (Å^2^)

**Table:**

	*x*	*y*	*z*	*U*_iso_*/*U*_eq_	
S1	0.37143 (5)	0.10537 (5)	0.24801 (4)	0.0559 (1)	
S2	0.72763 (5)	0.05293 (4)	0.29926 (4)	0.0542 (1)	
N1	0.44875 (14)	0.24375 (12)	0.38422 (10)	0.0392 (3)	
N2	0.76229 (15)	0.27317 (13)	0.37678 (11)	0.0446 (3)	
C1	0.25768 (16)	0.48352 (15)	0.31596 (13)	0.0404 (4)	
C2	0.20907 (19)	0.59310 (17)	0.37238 (15)	0.0501 (5)	
C3	0.2155 (3)	0.73893 (19)	0.2912 (2)	0.0663 (6)	
C4	0.2704 (3)	0.7760 (2)	0.1528 (2)	0.0733 (7)	
C5	0.3168 (3)	0.6681 (2)	0.09567 (17)	0.0680 (6)	
C6	0.3104 (2)	0.52245 (18)	0.17638 (15)	0.0525 (5)	
C7	0.25828 (17)	0.32402 (15)	0.40427 (14)	0.0447 (4)	
C8	0.50163 (17)	0.14720 (14)	0.32035 (13)	0.0408 (3)	
C9	0.8377 (2)	0.12518 (17)	0.38223 (16)	0.0541 (5)	
C10	0.56811 (17)	0.27834 (16)	0.44744 (13)	0.0431 (4)	
C11	0.80744 (19)	0.38248 (16)	0.24024 (13)	0.0449 (4)	
C12	0.7732 (2)	0.53983 (16)	0.23571 (14)	0.0488 (4)	
C13	0.8309 (2)	0.64508 (17)	0.09273 (15)	0.0560 (5)	
C14	0.8034 (3)	0.8041 (2)	0.0821 (2)	0.0821 (7)	
H2	0.17173	0.56829	0.46589	0.0601*	
H3	0.18275	0.81193	0.32993	0.0796*	
H4	0.27603	0.87410	0.09782	0.0878*	
H5	0.35270	0.69350	0.00210	0.0817*	
H6	0.34143	0.45000	0.13712	0.0630*	
H7A	0.20828	0.31702	0.49831	0.0537*	
H7B	0.17844	0.27896	0.38314	0.0537*	
H9A	0.82125	0.06214	0.47607	0.0649*	
H9B	0.97069	0.12161	0.33891	0.0649*	
H10A	0.52272	0.37597	0.45308	0.0516*	
H10B	0.55480	0.20912	0.53898	0.0516*	
H11A	0.73375	0.37322	0.18858	0.0538*	
H11B	0.93788	0.36004	0.19640	0.0538*	
H12A	0.84292	0.54959	0.28969	0.0586*	
H12B	0.64162	0.56520	0.27472	0.0586*	
H13A	0.96161	0.61717	0.05363	0.0672*	
H13B	0.75950	0.63540	0.03977	0.0672*	
H14A	0.84286	0.86437	−0.01109	0.1232*	
H14B	0.87606	0.81541	0.13228	0.1232*	
H14C	0.67380	0.83370	0.11828	0.1232*	

### Atomic displacement parameters (Å^2^)

**Table:**

	*U*^11^	*U*^22^	*U*^33^	*U*^12^	*U*^13^	*U*^23^
S1	0.0586 (2)	0.0591 (2)	0.0578 (2)	−0.0195 (2)	−0.0143 (2)	−0.0221 (2)
S2	0.0483 (2)	0.0444 (2)	0.0635 (3)	0.0008 (1)	−0.0102 (2)	−0.0197 (2)
N1	0.0350 (5)	0.0416 (6)	0.0381 (6)	−0.0065 (4)	−0.0072 (4)	−0.0118 (5)
N2	0.0389 (5)	0.0526 (7)	0.0390 (6)	−0.0087 (5)	−0.0112 (4)	−0.0100 (5)
C1	0.0315 (5)	0.0469 (7)	0.0422 (7)	−0.0041 (5)	−0.0099 (5)	−0.0150 (6)
C2	0.0469 (7)	0.0558 (9)	0.0505 (8)	−0.0020 (6)	−0.0139 (6)	−0.0230 (7)
C3	0.0691 (10)	0.0496 (9)	0.0874 (13)	0.0017 (7)	−0.0303 (9)	−0.0284 (9)
C4	0.0758 (11)	0.0464 (9)	0.0843 (14)	−0.0061 (8)	−0.0335 (10)	0.0005 (9)
C5	0.0694 (10)	0.0707 (11)	0.0470 (9)	−0.0072 (8)	−0.0176 (8)	−0.0018 (8)
C6	0.0538 (8)	0.0582 (9)	0.0446 (8)	−0.0032 (6)	−0.0131 (6)	−0.0189 (7)
C7	0.0341 (6)	0.0493 (8)	0.0443 (7)	−0.0075 (5)	−0.0021 (5)	−0.0148 (6)
C8	0.0415 (6)	0.0372 (6)	0.0365 (6)	−0.0109 (5)	−0.0062 (5)	−0.0052 (5)
C9	0.0422 (7)	0.0555 (9)	0.0545 (8)	−0.0025 (6)	−0.0164 (6)	−0.0076 (7)
C10	0.0434 (6)	0.0513 (8)	0.0331 (6)	−0.0106 (5)	−0.0087 (5)	−0.0115 (6)
C11	0.0448 (6)	0.0506 (8)	0.0380 (7)	−0.0121 (5)	−0.0067 (5)	−0.0139 (6)
C12	0.0507 (7)	0.0533 (8)	0.0435 (8)	−0.0120 (6)	−0.0096 (6)	−0.0169 (6)
C13	0.0636 (9)	0.0532 (9)	0.0485 (8)	−0.0137 (7)	−0.0140 (7)	−0.0116 (7)
C14	0.0893 (13)	0.0532 (10)	0.0946 (15)	−0.0123 (9)	−0.0219 (11)	−0.0158 (10)

### Geometric parameters (Å, °)

**Table:**

S1—C8	1.6776 (15)	C3—H3	0.9300
S2—C8	1.7470 (15)	C4—H4	0.9300
S2—C9	1.8479 (17)	C5—H5	0.9300
N1—C7	1.4760 (19)	C6—H6	0.9300
N1—C8	1.3246 (18)	C7—H7A	0.9700
N1—C10	1.4899 (18)	C7—H7B	0.9700
N2—C9	1.433 (2)	C9—H9A	0.9700
N2—C10	1.4326 (19)	C9—H9B	0.9700
N2—C11	1.4683 (18)	C10—H10A	0.9700
C1—C2	1.383 (2)	C10—H10B	0.9700
C1—C6	1.387 (2)	C11—H11A	0.9700
C1—C7	1.505 (2)	C11—H11B	0.9700
C2—C3	1.380 (3)	C12—H12A	0.9700
C3—C4	1.376 (3)	C12—H12B	0.9700
C4—C5	1.375 (3)	C13—H13A	0.9700
C5—C6	1.378 (3)	C13—H13B	0.9700
C11—C12	1.509 (2)	C14—H14A	0.9600
C12—C13	1.511 (2)	C14—H14B	0.9600
C13—C14	1.508 (3)	C14—H14C	0.9600
C2—H2	0.9300		
			
S1···H6	3.1400	H9A···S1^iii^	2.9300
S1···H7B	2.6000	H9B···S1^vi^	2.8800
S1···H9B^i^	2.8800	H9B···H11B	2.2900
S1···H5^ii^	3.1600	H10A···C1	2.7100
S1···H9A^iii^	2.9300	H10A···C2	2.9400
S1···H10B^iii^	3.1600	H10A···C12	2.7500
S2···H11A	2.9400	H10A···H7A	2.4600
N1···H11A	2.6800	H10A···H12B	2.2400
C6···C8	3.593 (2)	H10B···H9A	2.2800
C8···C11	3.374 (2)	H10B···S1^iii^	3.1600
C8···C6	3.593 (2)	H11A···S2	2.9400
C11···C8	3.374 (2)	H11A···N1	2.6800
C1···H12B	3.0800	H11A···C8	2.8300
C1···H10A	2.7100	H11A···H13B	2.5000
C2···H10A	2.9400	H11B···H9B	2.2900
C4···H14B^i^	3.0200	H11B···H13A	2.4400
C5···H13A^i^	3.0900	H11B···H13A^vii^	2.5700
C8···H11A	2.8300	H12A···H14B	2.5600
C10···H12B	2.8200	H12A···H2^v^	2.4800
C12···H10A	2.7500	H12B···C1	3.0800
C14···H4^iv^	3.0800	H12B···C10	2.8200
H2···H7A	2.3400	H12B···H10A	2.2400
H2···H12A^v^	2.4800	H12B···H14C	2.5700
H4···C14^iv^	3.0800	H13A···C5^vi^	3.0900
H4···H14A^iv^	2.4500	H13A···H11B	2.4400
H5···S1^ii^	3.1600	H13A···H11B^vii^	2.5700
H6···S1	3.1400	H13B···H11A	2.5000
H7A···H2	2.3400	H14A···H4^iv^	2.4500
H7A···H10A	2.4600	H14B···C4^vi^	3.0200
H7B···S1	2.6000	H14B···H12A	2.5600
H9A···H10B	2.2800	H14C···H12B	2.5700
			
C8—S2—C9	103.11 (7)	N1—C7—H7B	109.00
C7—N1—C8	122.01 (12)	C1—C7—H7A	109.00
C7—N1—C10	113.20 (11)	C1—C7—H7B	109.00
C8—N1—C10	124.74 (12)	H7A—C7—H7B	108.00
C9—N2—C10	109.50 (12)	S2—C9—H9A	109.00
C9—N2—C11	113.75 (12)	S2—C9—H9B	109.00
C10—N2—C11	115.24 (12)	N2—C9—H9A	109.00
C2—C1—C6	119.00 (14)	N2—C9—H9B	109.00
C2—C1—C7	120.70 (12)	H9A—C9—H9B	108.00
C6—C1—C7	120.29 (14)	N1—C10—H10A	109.00
C1—C2—C3	120.62 (15)	N1—C10—H10B	109.00
C2—C3—C4	119.82 (18)	N2—C10—H10A	109.00
C3—C4—C5	120.03 (18)	N2—C10—H10B	109.00
C4—C5—C6	120.31 (16)	H10A—C10—H10B	108.00
C1—C6—C5	120.21 (16)	N2—C11—H11A	109.00
N1—C7—C1	111.22 (11)	N2—C11—H11B	109.00
S1—C8—S2	112.92 (8)	C12—C11—H11A	109.00
S1—C8—N1	126.10 (11)	C12—C11—H11B	109.00
S2—C8—N1	120.94 (11)	H11A—C11—H11B	108.00
S2—C9—N2	113.13 (11)	C11—C12—H12A	109.00
N1—C10—N2	114.50 (11)	C11—C12—H12B	109.00
N2—C11—C12	114.63 (12)	C13—C12—H12A	109.00
C11—C12—C13	111.58 (12)	C13—C12—H12B	109.00
C12—C13—C14	113.99 (14)	H12A—C12—H12B	108.00
C1—C2—H2	120.00	C12—C13—H13A	109.00
C3—C2—H2	120.00	C12—C13—H13B	109.00
C2—C3—H3	120.00	C14—C13—H13A	109.00
C4—C3—H3	120.00	C14—C13—H13B	109.00
C3—C4—H4	120.00	H13A—C13—H13B	108.00
C5—C4—H4	120.00	C13—C14—H14A	109.00
C4—C5—H5	120.00	C13—C14—H14B	109.00
C6—C5—H5	120.00	C13—C14—H14C	109.00
C1—C6—H6	120.00	H14A—C14—H14B	109.00
C5—C6—H6	120.00	H14A—C14—H14C	109.00
N1—C7—H7A	109.00	H14B—C14—H14C	109.00
			
C9—S2—C8—S1	178.01 (8)	C9—N2—C11—C12	−165.23 (13)
C9—S2—C8—N1	−0.05 (12)	C10—N2—C11—C12	67.14 (17)
C8—S2—C9—N2	−29.53 (12)	C6—C1—C2—C3	−1.0 (2)
C8—N1—C7—C1	−108.21 (14)	C7—C1—C2—C3	177.50 (17)
C10—N1—C7—C1	74.18 (14)	C2—C1—C6—C5	1.0 (2)
C7—N1—C8—S1	2.68 (19)	C7—C1—C6—C5	−177.45 (17)
C7—N1—C8—S2	−179.54 (10)	C2—C1—C7—N1	−114.76 (15)
C10—N1—C8—S1	−180.00 (10)	C6—C1—C7—N1	63.68 (17)
C10—N1—C8—S2	−2.21 (18)	C1—C2—C3—C4	0.1 (3)
C7—N1—C10—N2	−147.79 (12)	C2—C3—C4—C5	0.7 (4)
C8—N1—C10—N2	34.68 (18)	C3—C4—C5—C6	−0.6 (4)
C10—N2—C9—S2	62.06 (13)	C4—C5—C6—C1	−0.2 (3)
C11—N2—C9—S2	−68.49 (15)	N2—C11—C12—C13	177.42 (13)
C9—N2—C10—N1	−65.65 (15)	C11—C12—C13—C14	−178.86 (16)
C11—N2—C10—N1	64.09 (17)		

Symmetry codes: (i) *x*−1, *y*, *z*; (ii) −*x*+1, −*y*+1, −*z*; (iii) −*x*+1, −*y*, −*z*+1; (iv) −*x*+1, −*y*+2, −*z*; (v) −*x*+1, −*y*+1, −*z*+1; (vi) *x*+1, *y*, *z*; (vii) −*x*+2, −*y*+1, −*z*.

### Hydrogen-bond geometry (Å, °)

**Table:**

*D*—H···*A*	*D*—H	H···*A*	*D*···*A*	*D*—H···*A*
C7—H7B···S1	0.97	2.60	3.0978 (16)	112
C12—H12B···CgA	0.97	2.96	3.7937 (19)	145
